# An overview of orthodontic malpractice liability based on a survey and case assessment review

**DOI:** 10.4317/jced.59785

**Published:** 2022-09-01

**Authors:** Hamid Pour, Karthikeyan Subramani, Richard Stevens, Pramod Sinha

**Affiliations:** 1 Roseman University of Health Sciences, College of Dental Medicine, Henderson, NV, USA

## Abstract

**Background:**

The purpose of this survey study and case review was to identify 1) the common causes related to filing a malpractice claim against an orthodontist and, 2) the factors mitigating against a potential malpractice claim in the United States (U.S). The objectives of the case review were to examine the current state of orthodontic malpractice litigation from a cause and mitigating point of view.

**Material and Methods:**

Data for this research was collected and reviewed using the following two methods: 1) A survey questionnaire on aspects of malpractice liability was electronically distributed to 2,241 active U.S. members of the American Association of Orthodontists (AAO). 2) Legal cases were reviewed on the online legal research database Lexis Advance Research, and 35 cases were analyzed.

**Results:**

Survey questionnaire results and legal case review results are as follows: 1) 77 orthodontists completed the survey. 9.1% of the respondents reported a malpractice claim having been filed against them with periodontal issues accounting for most of the claims. Survey participants reported good doctor-patient communication as being the most relevant contributory factor and most relevant mitigating factor in malpractice claims. 2) Negligence is the main reason patients sue a doctor for clinically related litigation, and failure to obtain a proper informed consent from the patient is the main cause of action for non-clinically related litigation.

**Conclusions:**

Most respondents reported doctor-patient communication, periodontal issues, and a lack of informed consent as the main triggering elements of a lawsuit, which is similar to other studies and case review analysis. Good doctor-patient rapport was ranked as being most helpful in mitigating a potential claim, which is also similar to other studies. Another aspect of the survey questionnaire that was evaluated was whether a non-orthodontist can provide expert testimony against an orthodontist, with most respondents reporting that this is not possible. It has been ruled, though, that a general dentist can be an expert witness and provide expert testimony against an orthodontist in a lawsuit depending on the circumstances. Negligence was the most common cause of clinically related orthodontic litigation, and a failure of the practitioner to obtain a proper informed consent from the patient was the most common cause of non-clinically related orthodontic litigation.

** Key words:**Orthodontist, malpractice, legal, lawsuit, liability, medicolegal.

## Introduction

There is a lack of information on the various types of malpractice litigation in orthodontics. There are several reasons why a patient can file a malpractice litigation against an orthodontic provider. However, there are similar complaints or litigation that tend to repeat over time. Mistakes that are repeated are unnecessary, expensive to both the orthodontist and patients and can be detrimental for both parties. Alternatively, there can be new complaints filed against orthodontists due to new or changing technology. It is necessary to investigate the current status of malpractice claims in orthodontics in the United States (U.S).

The U.S. currently ranks highest in health care spending among the world’s developed nations. Health care spending was $2.3 trillion in 2007 and $3.5 trillion in 2017, which is an increase of over 50% from 2007 to 2017. Defensive medicine is one of the main reasons for the increasing cost of health care (1). Defensive medicine is a safeguard from litigation where physicians prescribe diagnostic tests or provide medical treatments that depart from normal practice of medicine (1). It has been show that “there is a statistically significant correlation between a specialists’ concerns regarding potential medico-legal disputes and the choice of defensive medical procedures” (1).

Some studies show that the number of malpractice claims have decreased since 2001, however malpractice claims are still common in the U.S. with the average cost of these claims having been shown to increase (1). Malpractice insurance premiums have also increased accordingly, which can be attributed to the high cost of malpractice claims. This rising cost of business will, in turn, affect a doctor’s income. While malpractice cases for doctors with an MD or DO degree have been studied in recent years, studies on dental malpractice disputes are lacking (1). There is even less information on orthodontic malpractice claims and, although studies on orthodontic claims are minimal, history has shown that an orthodontic claim can be substantial (2).

Negligence is a major cause of dental malpractice suits. A doctor can be sued for negligence when the doctor guarantees certain results that were promised orally, in writing, or by implication, when the doctor was negligent in their treatment of the patient, and in failing to obtain a proper informed consent from the patient.

Professional negligence is defined as “a negligent act or omission to act by a health care provider in the rendering of professional services, which act or omission is the proximate cause of a personal injury or wrongful death” (3). Applying this definition to a legal case poses difficulties because “additional claims often arise out of the same facts as a professional negligence claim, including claims for battery, products liability, premise liability, fraud, breach of contract, and intentional or negligent infliction of emotional distress” (3). Hence, the scope and meaning of the phrase “based on professional negligence” varies (3). Furthermore, dental malpractice is defined as “failure on the part of a dentist to exercise the degree of care, diligence and skill ordinarily exercised by dentists in good standing in the community in which he or she practices” (4).

Literature review of orthodontic litigation can be divided into studies solely in orthodontics and studies in the dental field that includes information on orthodontics. These two categories can be subdivided into studies done within the U.S. and studies done abroad.

The only study done directly for orthodontics in the U.S. was conducted in 1990 via a survey that was sent to practicing orthodontists in the State of Florida (5). Most orthodontists perceived the malpractice environment as threatening, although most orthodontists who held this perception only had “passive” malpractice experience where they either talked, listened, or heard of malpractice actions against orthodontists and rarely had “active” experiences in a malpractice action, such as being an expert witness, being involved in a lawsuit, or ever having to consult with an attorney. This perception of a threatening legal environment did impact how orthodontists treat and manage their patients by taking more defensive and conservative therapies, such as taking frequent radiographs, performing more TMJ examinations, treatment planning fewer extractions, delegating less duties, obtaining more second opinions, and more paperwork and note taking (5).

There have been more studies on orthodontic legal environment abroad with studies in Israel, Brazil and England (6-8). In Israel, claims where periodontal problems either preceded or followed orthodontic treatment and resulted in a legal decision between 2005-2018 were analyzed (7). It was shown that 97% of the claims analyzed in the study were complications caused by periodontal disease or aggravation. Periodontal problems can lead to additional attachment loss during orthodontic treatment and, since there is an increasing number of adult patients seeking orthodontic treatment during the past 10 years and older patients have a greater chance of having underlying periodontal disease as opposed to younger patients, orthodontic therapy could worsen the periodontal disease in these older patients. Controlling biofilm and having a 1-3 month periodontal maintenance program during active orthodontic treatment becomes essential. Taking comprehensive records before, during, and after treatment, obtaining a restorative, prosthodontic and periodontal dental clearance from patients, discussing the treatment plan in detail, and obtaining a proper informed consent aids in avoiding any potential litigation (7).

A study in Brazil showed that orthodontists have little knowledge regarding their legal rights and obligations as healthcare providers (6). Law professionals, orthodontists, and orthodontic patients were interviewed separately, and the results showed a lack of transparent conversation between orthodontists and patients. There was a failure of a proper patient-professional relationship, which was a triggering element for legal compensation actions or as a real background for initiating lawsuits. It was also shown that there are insufficient graduate schools in Dentistry with “Juridical Deontology in their curriculum,” and a lack of coursework or lectures on this topic for orthodontists to practice in a preventive manner (6). Standardized and customized service contracts drafted by a specialized lawyer, coupled with complete orthodontic records, can mitigate this shortcoming to defend orthodontists (6).

In England, 2.9% of all the cases addressed by the Professional Conduct Committee of the General Dental Council from 2005-2010 were related to orthodontics (8). This does not reflect litigated cases that do not progress to a disciplinary hearing of the General Dental Council, since such information was not available.8 The study showed that patients are generally less likely to sue their orthodontist when a good personal relationship exists, and patients who are upset and feel resentment against their orthodontist will usually litigate. A key risk management feature is to establish a pleasant, friendly, and empathetic relationship with patients, with orthodontic risk assessment being better understood by classifying the doctor-patient relationship into three periods: pre-treatment, treatment and post-treatment (8).

Establishing a good professional relationship with the patient while ensuring that the relationship and level of conversation matches the patient’s age is a common theme throughout the entire orthodontic experience regardless of the treatment period. Furthermore, being empathetic, smiling, being pleasant, and communicating with the patient is also important throughout the entire orthodontic experience (8).

Data from general dentistry malpractice claims can provide some insight on orthodontic malpractice claims and provide an overview of trends in the entire dental field. A survey of all dental malpractice claims in the U.S. that closed in 1970, which included incidents occurring from 1959-70, was conducted by the Secretary’s Commission on Medical Malpractice, U.S. Department of Health, Education, and Welfare (HEW). Not surprisingly, specialists such as oral surgeons were at higher risk of malpractice claims than general practitioners. The amount of damages paid to claimants for dental cases was about 1/3rd of what was paid on claims that involved physicians or medical specialists. In 1970 dollars, the median award to a plaintiff for a dental malpractice claim was $750, and 95% of the awards were under $5,000 (9).

Claims were also broken down by specialty, with claims against orthodontists accounting for 5.1% of the claims closed in 1970 (9). However, orthodontists represented 4.5% of the practicing dentists at that time and, hence, there were more claims than the population of orthodontists at that time (9). The study also showed that the likelihood of a claim being filed against a dentist dropped after the age of 35 and remained low until rising at age 45. Established patients were also less likely to initiate a malpractice claim against their dentist than were new patients, with more than 75% of the filed claims being from patients who were being treated by the practitioner for less than 1 year, and 84% of the claims filed were from patients who had been treated by the practitioner for less than 2 years (9). Less than 10% of the claims filed were from patients who had been seeing the same dentist for 5 years or longer (9).

Dental malpractice claims were not directly studied again until a survey of dentists nationwide was done to collect the number of patient complaints from 1988-92 (10). The specialties of dentistry with the most number of complaints were oral surgery, which accounted for 21.9% of the dental claims, and fixed prosthodontics at 19.5%. Orthodontics only had two claims that were filed during this period. The study showed that the number of total filed claims and the percentage of closed claims resulting in payment increased as compared to the 1970 HEW study (10). Oral surgery had the largest proportion of claims with more claims being filed in fixed prosthodontics, endodontics, periodontics and restorative dentistry (10).

A study in Rome, Italy analyzed dental malpractice between 2001 to 2015 and showed that dental malpractice claims have decreased with the 2013 case number being 1/3rd of the 2002 case number (11). Dental claim verdicts showed a general downward trend (11). The article argues two main reasons for the decrease in the number of dental claims: 1) dentists are more aware and mindful in approaching patients, and 2) out of court settlements have increased. The second argument is difficult to verify due to the lack of data released by insurance companies (11). Data could possibly be obtained directly from dentists via a survey to circumvent the obstacle presented by insurance companies.

Two studies from Iran conducted a retrospective study of dental malpractice claims, with one study collecting data from claims in Tehran, Iran between 2002 and 2006 and another study collected data in Kerman, Iran from 2000-2011 (12,13). The majority of complaints were in fixed prosthodontics and oral surgery with both studies differentiating between clinical and non-clinical malpractice claims (12,13). Clinical malpractice accounted for 67.2% and non-clinical cases accounted for 32.8% of the malpractice claims in Tehran, with 56.7% of the complaints in the Kerman study being clinical in nature and 40% non-clinical (12,13). Non-clinical claims included advertisement violations, practicing without a license, sexual harassment and swindling (13). The study did show few complaints in orthodontics (13). However, the study did not specify if the complaints were against orthodontists alone or orthodontists and general dentists practicing orthodontics (13). Complaints in orthodontics accounted for 10.5% of the malpractice claims in Tehran and 3.1% in Kerman (12,13). The most frequent causes of orthodontic complaints were treatments that were below the standard of care, dissatisfaction with the treatment outcome, misdiagnosis, inappropriate treatment, insufficient attention to the patient in relation to treatment, and lack of sufficient skills (12,13).

Litigation in Iran is increasing, contrary to the decreasing number of claims in Rome, Italy (13). However, no data was given as to the trend in dental malpractice claims in Tehran during this period (13). The authors proposed that litigation in Iran has been increasing due to an increase in the number of practicing dentists, which resulted in an increased number of treatments provided and, in turn, the increased treatments have increased the risk of malpractice (13). In addition, the authors claim that the expanding population of patients is becoming more knowledgeable and aware of its rights and is taking action by filing complaints through the courts (13).

More studies have been done abroad on dental malpractice claims than in the U.S. These studies from abroad have shown mixed results as far as trends in the number of litigations. Some show a decrease in the number of dental claims (11), while others show an increase in dental claims (12,13). However, all have been consistent in that oral surgery comprises most of the claims made while orthodontics is on the low end (11-14). The purpose of this survey study and case review was to identify 1) the common causes related to filing a malpractice claim against an orthodontist and, 2) the factors mitigating against a potential malpractice claim in the U.S. The objectives of the case review were to examine the current state of orthodontic malpractice litigation from a cause and mitigating point of view.

## Material and Methods

Data for this research was collected and reviewed using two different methods.

Method 1 for Survey Questionnaire: A survey questionnaire was developed on a secure online survey platform, Qualtrics (www.qualtrics.com). A pilot study was conducted among the orthodontic faculty of Roseman University of Health Sciences prior to distribution of the survey. The study was approved by the Roseman University of Health Sciences Institutional Review Board and subsequently reviewed and approved by American Association of Orthodontists (AAO) Partners in Research. The link to the survey and a cover letter explaining the objectives were distributed electronically through email to a random sample of 2,241 active U.S. AAO members, including orthodontic faculty. Retired orthodontists and orthodontic residents were excluded from the study. A reminder email was sent 2 weeks later, and data was collected over a 1-month period following which the survey was closed.

Collected data were analyzed with IBM® SPSS® version 27. Descriptive statistics were generated to analyze the frequency of response regarding factors that contribute to a patient filing a malpractice claim against an orthodontist in terms of 1) practitioner’s information 2) whether a claim has been filed against the orthodontist, 3) factors that contribute to malpractice claims against orthodontists, 4) practice management methods to mitigate patient claims, and 5) increasing knowledge and awareness of common malpractice claims to mitigate any potential damage.

Method 2 for Legal Cases: Legal cases, including jury verdicts and settlements, were reviewed on the online legal research database Lexis Advance Research, which is an online search engine with cases being found through keywords. The case review search was limited by publication dating back to the year 2000. This was done because the introduction of a variety of computing, imaging, and milling techniques, robotic technologies, and aligners have brought about an esthetic revolution (15,16). Therefore, the orthodontic field has undergone a considerable shift in techniques and treatment.

All cases involving orthodontic providers, orthodontic practices and corporations, and orthodontic treatments were included. Each case was read to determine its relevancy to orthodontics. Then, the data from orthodontic related cases were systematically assessed and recorded for certain characteristics, including the state in which the trial had proceeded, chronologic analysis, award payouts and jury verdicts. Data was further categorized into its underlying etiology to characterize the circumstances that led to the lawsuit, and descriptive Tables were used to explain the frequency distribution of the various causes of action. 35 cases were included in this study.

The following terms were considered: orthodontics, orthodontist, jurisprudence, lawsuit, legal action, litigation, malpractice, sued, medicolegal or medic-legal or medico legal, dentolegal or dento-legal or dento legal, Invisalign, clear aligner, and braces.

Search Examples are the following:

1) ((“Orthodontists”[Mesh]) OR “Orthodontics”[Mesh]) AND ((“Liability, Legal”[Mesh] OR “Jurisprudence”[Mesh])) NOT Jerrold L[AU]

2) ((orthodontics) OR orthodontist)) AND (((legal) OR lawsuit) OR malpractice))) NOT age

3) ((orthodontics) OR orthodontist)) AND (((legal) OR lawsuit) OR malpractice))) NOT Jerrold L[AU]

4) ((orthodontics) OR orthodontist)) AND (((legal) OR lawsuit) OR malpractice))) NOT “Int J Legal Med”[jour]

5) ((orthodontics) OR orthodontist)) AND (((medico legal) OR medicolegal) OR medico-legal)))

6) (((((orthodontics) OR orthodontist) OR braces) OR Invisalign) OR clear aligner))))) AND (((((legal) OR lawsuit) OR malpractice) OR litigation) OR sued)))))

The inclusion criteria included all orthodontic malpractice litigation cases reported on Lexis, all orthodontic providers irrespective of specialty status, and litigation with and without jury verdicts and settlements. The exclusion criteria included non-orthodontic treatments, orthodontic litigation not termed as malpractice on Lexis, duplicate cases, and orthodontic cases between DSOs, such as for security fraud or patent infringement.

## Results

**Figure 1:**
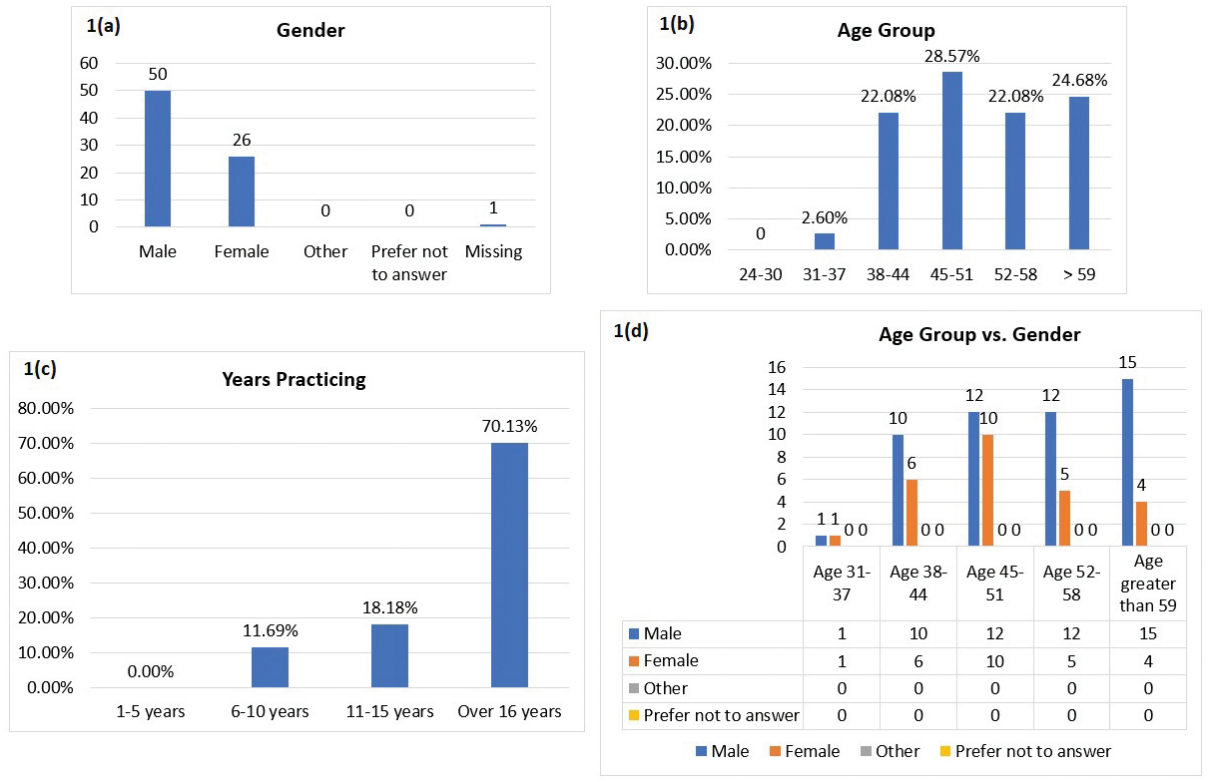
a. Gender of respondents. 50 males and 26 female orthodontists participated in the survey with 1 missing. b. Age group of respondents. The highest number of responses came from the age group of 45-51 years (28.6%). c. Respondent practicing years. Most responses were from orthodontists practicing for more than 16 years. d. Cross tabulation of Age Group and Gender. Most responses were from males over 59 years of age.

**Figure 2:**
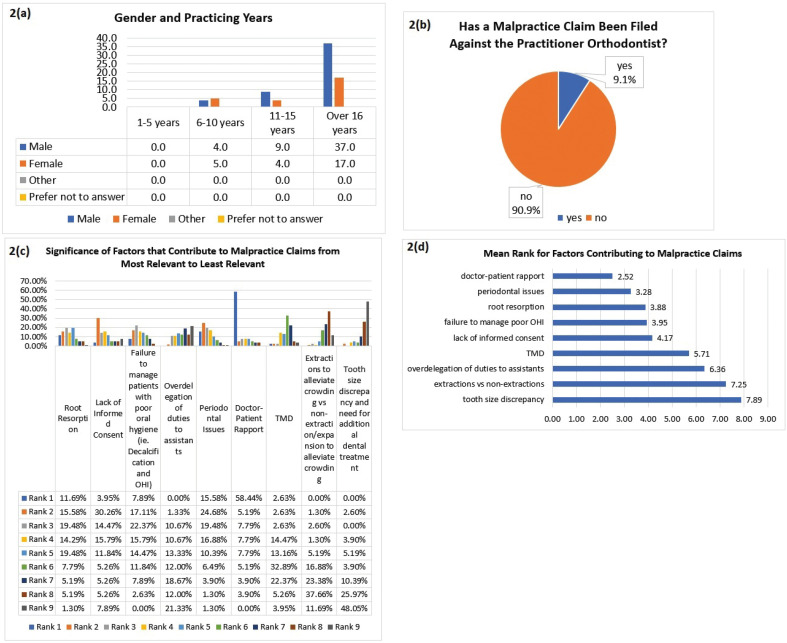
a. Cross tabulation of Gender and Practicing Years. Most responses were from males practicing for more than 16 years. b. Percentage of orthodontists that have had a malpractice claimed filed against them. Seven (9.1%) of the orthodontists that participated in this survey reported a malpractice claim has been filed against them, while the other 90.9% did not. c. Significance of factors that contribute to a patient filing a malpractice claim against an orthodontist. Participants were asked to rank factors from 1 being the most relevant to 9 being the least relevant in contributing to a malpractice claim in orthodontics. 58.44% of participants ranked doctor-patient rapport as being most relevant and contributory to a patient filing a claim against an orthodontist. d. Mean rank of contributing malpractice factors. The mean rank for periodontal issues is 3.28 ahead of lack of informed consent at 4.17.

-Results from Survey:

A total of 77 responses were recorded with no exclusions or eliminations. The following groups were found to have higher responses: (a) males (Fig. 1a); (b) practitioners within the 45-51 year age group (Fig. 1b); (c) clinicians who have been in practice for more than 16 years (Fig. 1c); (d) males over 59 years of age (Fig. 1d); and (e) males practicing for more than 16 years (Fig. 2a).

Seven (9.1%) of the orthodontists that participated in this survey reported that a malpractice claim has been filed against them (Fig. 2b). All seven were practicing for more than 16 years, and 6 out of the 7 orthodontists were males. Further, 2 were in the age group of 45-51 years, 3 in 52-58 years, and 2 greater than 59 years. The questionnaire revealed the reasons for a malpractice claim being filed against them. Periodontal issues was the most common reason for a claim being filed, with 3 responses, followed by 1 response equally among: a) root resorption, b) an ankylosed impacted tooth that was scheduled for extrusion but did not extrude, which lengthened the treatment time and caused untoward problems of the remaining dentition, c) colleague ethics, and d) loss of a tooth due to periodontal and endodontic issues.

Participants were then asked to rank factors that contribute to malpractice claims against orthodontists. Doctor-patient rapport was ranked as being most relevant and contributory to a patient filing a claim against an orthodontist 58.44% of the time (Fig. 2c). Lack of informed consent was ranked as the second most contributory factor 30.26% of the time, with periodontal issues ranking second 24.68% of the time. The mean rank for periodontal issues, however, is 3.28 (Fig. 2d).

**Figure 3:**
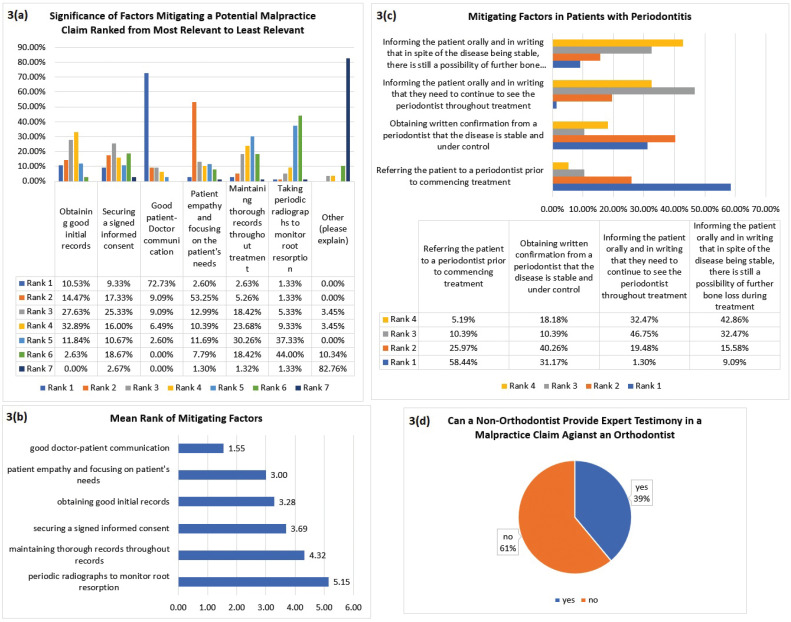
a. Significance of factors mitigating against a potential malpractice claim in orthodontics. Participants were asked to rank factors from 1 being the most relevant to 7 being the least relevant in mitigating against a potential malpractice claim in orthodontics. Good doctor-patient communication was ranked as the most contributory factor 72.73% of the time. b. Mean rank of factors that mitigate the filing of a potential malpractice claim against an orthodontist. Good doctor-patient communication was ranked as the more relevant factor in mitigating against a potential malpractice claim with a mean rank of 1.55. c. Significance of factors that mitigate against a malpractice claim in patients with periodontitis. Participants were asked to rank factors from 1 being the most relevant to 4 being the least relevant. Referring a patient to the periodontist prior to commencing treatment was ranked as the most relevant mitigating factor. d. Percentage of orthodontists reporting on non-orthodontist expert testimony. 61.0% of participants reported that a non-orthodontist cannot provide expert testimony against an orthodontist in a malpractice claim.

Good doctor-patient communication was ranked as the most relevant factor in mitigating a potential malpractice claim 72.73% of the time (Fig. 3a) with a mean rank of 1.55 (Fig. 3b). Referring the patient to a periodontist prior to commencing treatment ranked as the most relevant mitigating factor in patients with periodontitis at 58.44% (Fig. 3c). An option for participants to explain other mitigating factors was ranked as the least relevant factor with a minimum ranking of 3 and a maximum ranking of 7. Other mitigating factors include: a) avoiding monetary disputes (ranked as 3rd most relevant by one participant), b) refund (ranked as 4th most relevant by one participant), c) discussing all treatment options, record keeping and missed/failed appointments, and working with a general dentist to manage cases each being ranked as the 6th most relevant separately, d) being honest, continuing education, not sending patients to collections, and proper communication with the patient and dentist each being ranked as the least relevant mitigating factor. One participant commented that all factors are relevant equally. Other was ranked as the least relevant mitigating factor by 19 participants without any explanation.

**Figure 4:**
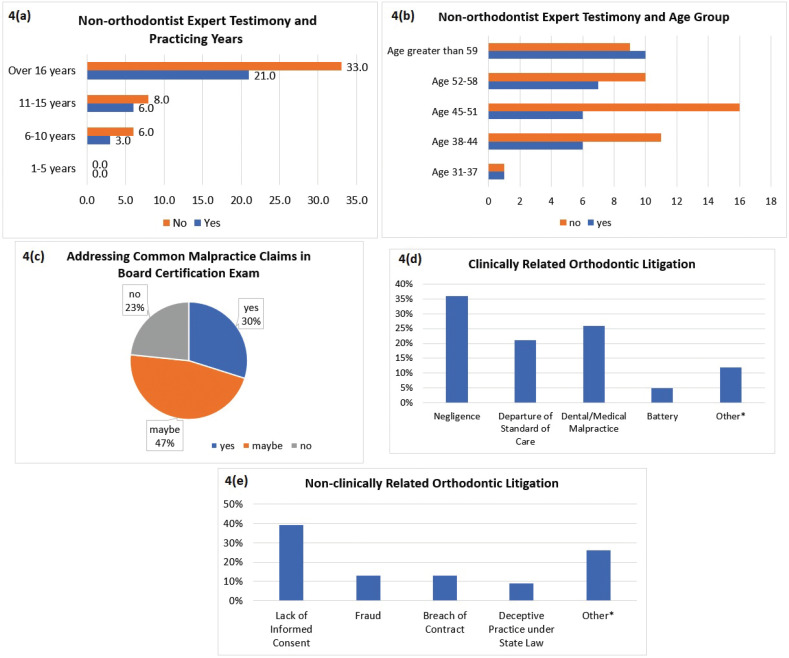
a. Cross tabulation of non-orthodontist expert testimony and practicing years. A majority of participants reported that a non-orthodontist cannot provide expert testimony in a malpractice claim against an orthodontist throughout all practicing years. b. Cross tabulation of non-orthodontist expert testimony and age group. A majority of participants aged 38-58 reported that a non-orthodontist cannot provide expert testimony against an orthodontist. The age group 31-37 was evenly split. A majority of participants in the age group greater than 59 reported that a non-orthodontist can provide expert testimony. c. Percentage of orthodontists reporting on whether addressing common malpractice claims in board certification exams is beneficial. 46.8% of participants were unsure and reported “maybe”. d. Categorization of the causes of action in clinically related orthodontic litigation in percentage. The main cause of action in clinically related orthodontic litigation was negligence at 36% of the cases reviewed. (Other* - Misdiagnosis/mistreatment, Intentional infliction of emotional distress, Neglect, Wrongful death, Violation of personal liberty). e. Categorization of the causes of action in non-clinically related orthodontic litigation in percentage. 39% of the cases categorized under non-clinically related orthodontic litigation showed that practitioners were sued failing to obtain a proper informed consent from the patient. (Other* - Discrimination, Abandonment, Anticipatory repudiation, Tortious interference of contract, Unjust enrichment, Civil conspiracy).

When participants were asked whether a non-orthodontist can provide expert testimony against an orthodontist during a malpractice claim, 61% of the participants said no (Fig. 3d). This question was cross-tabulated with the number of years the participant was practicing, and a majority of the participants reported that a non-orthodontist cannot provide expert testimony in a malpractice claim against an orthodontist throughout all practicing years (Fig. 4a). When the non-expert testimony data was cross-tabulated with age group, a majority of participants aged 38-58 reported that a non-orthodontist cannot provide expert testimony against an orthodontist. The age group 31-37 was evenly split, and a majority of participants in the age group greater than 59 reported that a non-orthodontist can provide expert testimony (Fig. 4b). Finally, 46.8% of the participants were unsure whether addressing common malpractice claims in board certification exams was beneficial (Fig. 4c).

-Review of Legal Cases

The case review search was limited by publication to the U.S. dating back to the year 2000. This was done because the introduction of a variety of computing, imaging and milling techniques, robotic technologies, and aligners have brought about an esthetic revolution which has limited the case review by publication dating back to the year 2000 (15,16). Therefore, the orthodontic practice has undergone a significant shift in tools and techniques.

The results of 35 legal cases reviewed from various US jurisdictions were divided into clinically related and non-clinically related orthodontic litigation and summarize the legal cases against orthodontists in court. Negligence is the most common reason orthodontists have been sued for clinically related litigation (Fig. 4d). Failure to obtain a proper informed consent is the number one reason orthodontists are sued for non-clinically related litigation (Fig. 4e). The causes of action listed under other, for both diagrams, have shown up only once.

## Discussion

-Discussion from Results of Survey

Our study found that 9.1% of the survey participants had to defend a malpractice claim (Fig. 2b). There may be a discrepancy in how many claims were reported. This number could possibly be higher, due to some practitioners being apprehensive in reporting a malpractice claim. Obtaining data from insurance companies is also difficult. The survey asked about filed claims, and there are probably a greater number of patient-doctor disputes without an actual claim being filed. An opportunity exists to gain more insight into patient management by inquiring about disputes where no claim was filed.

However, numbers are higher in this study when compared to most numbers found in other surveys. In England, 2.9% of all the cases addressed by the Professional Conduct Committee of the General Dental Council in England from 2005-2010 were related to orthodontics (8). A US study from 1970 reported that only 5.1% of claims closed in 1970 were against orthodontists (9). Two studies from Iran reported that 3.1% of malpractice claims in Kerman were in orthodontics, however, 10.5% of malpractice claims in Tehran were in orthodontics (12,13). However, there are discrepancies between this survey and studies done domestically and internationally.

Numbers reported in England only reflect litigated cases that do not progress to a disciplinary hearing of the General Dental Council making it likely that claims in England are greater than 2.9% (8). Also, there were more per capita claims against US orthodontists in 1970, with orthodontists only representing 4.5% of the practicing dentists at that time (9). Furthermore, differences in legal systems between countries will lead to varying data since lawsuits may be easier to file in some countries.

When comparing malpractice claims with the age of the practitioner, all claims were against orthodontists over 45 years of age with numbers being split almost equally between age groups over 45 years. Filed claims becomes more likely with increased age. Compared to the US study done in 1970, claims filed against a dentist dropped after the age of 35 and remained low until rising at age 45, which is similar to numbers reported in the survey. However, the 1970 study showed that established patients were less likely to initiate a malpractice claim against their dentist than new patients, with more than 75% of the claims filed being from patients that were treated by a practitioner for less than 1 year, 84% from less than 2 years, and this number dropped to less than 10% when a patient was seeing the same dentist for 5 years or longer (9). Our survey contrastingly shows that claims increased with a practitioners years of practice, since all of the malpractice claims reported were against practitioners who were practicing for more than 16 years.

The most common causes for complaints in orthodontics in international studies were similar to those reasons reported by participants. Frequent errors that led to malpractice claims internationally were inappropriate procedure, misdiagnosis, failure to treat properly, lack of informed consent, inadequate precautions to prevent injury, wrong treatment, root resorption, lack of sufficient skill, and insufficient patient attention in relation to treatment (12,13). Reasons reported by participants of an ankylosed impacted tooth that did not extrude, which lengthened treatment time and caused untoward problems of the remaining dentition, and loss of teeth due to periodontal and endodontic issues, can be attributed to either inappropriate procedure, failure to treat properly, lack of sufficient skill or insufficient patient attention in relation to treatment.

Doctor-patient rapport was ranked as being most relevant and contributory to a patient filing a claim against an orthodontist 58.44% of the time (Fig. 2c). Lack of informed consent was ranked as the second most contributory factor 30.26% of the time, with periodontal issues being ranked second 24.68% of the time. The five main factors that contribute to malpractice claims per the participants are: 1) doctor-patient rapport; 2) periodontal issues; 3) root resorption; 4) failure to manage poor oral hygiene; and 5) lack of informed consent (Fig. 2d). Lack of informed consent is the most common non-clinically related orthodontic litigation. There is overlap between negligence and not obtaining proper informed consent, which further emphasizes the need for practitioners to have good records and a proper informed consent to aid in their defense of a legal claim (17). The main factor mitigating a potential malpractice claim is maintaining good doctor-patient communication (Fig. 3b), and referring a patient with periodontitis to a periodontist prior to commencing treatment (Fig. 3c).

International studies showed a lack of transparent conversation between orthodontists and patients as a triggering element for legal compensation actions or as a real background for initiating lawsuits (6). Orthodontists should establish a pleasant, friendly and empathetic relationship with their patients to mitigate risk, since patients are less likely to sue their orthodontist when a good personal relationship exists. Patients who are upset and feel resentment against their orthodontist are more likely to litigate (8).

To avoid potential litigation in patients with periodontitis, controlling biofilm and having a 1-3 month periodontal maintenance program during active orthodontic treatment, especially in older patients, in addition to obtaining a periodontal clearance is essential (7). Taking comprehensive records, obtaining a dental clearance from patients, discussing the treatment plan in detail with an explanation of all the benefits and complications of the proposed treatment, and obtaining a proper informed consent are additional factors in avoiding litigation (6,7).

Most of the participants reported that a non-orthodontist cannot provide expert testimony against an orthodontist in a malpractice claim (Fig. 3c). It was ruled in a Nevada Court that a general dentist can be an expert witness and provide expert testimony against an orthodontist. This was allowed by the Nevada Court since the general dentist was testifying as to whether the orthodontist exercised due care in general orthodontic practices. This included testimony on the patient’s periodontal condition during treatment, if proper oral hygiene instructions were provided to the patient by the doctor or staff, and whether extractions of some of patient’s teeth prior to treatment was required. The general dentist was not providing an opinion specific to lingual braces and, hence, a non-orthodontist expert testimony was allowed (4).

The questionnaire could define non-orthodontist more clearly. Non-orthodontist could include a lay person, a healthcare provider, or a non-orthodontist dentist. Hence, there may be a discrepancy as participants may have interpreted non-orthodontist differently.

46.8% of the participants were unsure whether addressing common malpractice claims in board certification exams was beneficial (Fig. 4c). In Brazil, three groups of individuals, which consisted of law professionals, orthodontists, and orthodontic patients, were interviewed separately to prove that orthodontists have little knowledge regarding their legal rights and obligations as healthcare providers. The answers of the interviews showed a lack of transparent conversation between orthodontists and patients, which hinders establishing a proper patient-professional relationship and can trigger a legal compensation action. The study showed that there is insufficient lectures or coursework in dental programs to provide orthodontist with sufficient knowledge in patient management (6).

-Discussion from Review of Legal Cases

Cases are not always decided on their merits, and cases can be dismissed for a variety of reasons without a decision being made on whether the orthodontist was at fault or not. For example, cases can be dismissed as being barred by the state’s statute of limitations. However, the reason for the patient suing the orthodontist is still available in the court case. Negligence is the most common clinically related litigation. Negligence encompasses many different causes of action. An orthodontist can be held negligent for failing to refer a case to a specialist and when negligently advising a doctor in another state during treatment (18-20). Negligence also covers patients that lose their teeth during treatment whether from root resorption or from the wrong tooth being extracted. Patients have lost their teeth due to root resorption from extensive orthodontic treatment and subsequently received implant restorations. Patients have also lost teeth from more minor treatments, such as clear aligner treatment to close a diastema between the upper central incisors where initial patient records were not taken and only a visual exam was completed. The treatment resulted in loose and abscessed teeth with alveolar bone loss, and the patient being awarded $154,644.18 (21-23).

Negligence can also include failing to advise a patient on the treatment alternatives, which overlaps with a lack of informed consent. A patient whose lower incisor was extracted due to crowding was not informed of the risk of losing the remaining lower anterior teeth. The patient eventually lost the remaining lower incisors, and the doctor did not advise the patient of the risk nor did the doctor advise the patient of other treatment alternatives and their respective risks and advantages to enable the patient to make an informed decision. This highlights how different causes of action tend to overlap (24).

An orthodontist can be held liable for negligence based on expert testimony from a general dentist. The Court in Nevada ruled that a general dentist can testify as an expert against an orthodontist for treatment done by the orthodontist. A Nevada orthodontist was sued for injuries sustained by a patient during the course of lingual braces treatment. The general dentist had experience treating patients with Invisalign but not with a lingual braces system. However, the general dentist was testifying as to whether the orthodontist exercised due care in general orthodontic practices, such as testifying as to the patient’s periodontal condition during treatment, if proper oral hygiene instructions were provided to the patient by the doctor or staff, and whether extraction of some of the patient’s teeth prior to treatment was required. Hence, the Court ruled that the general dentist was qualified as an expert witness, since the general dentist’s opinions were not specific to lingual braces. The patient was eventually awarded $472,380.11 (4).

Lack of informed consent is the most common non-clinically related orthodontic litigation. There is overlap between negligence and not obtaining proper informed consent, which further emphasizes the need for practitioners to have good records and a proper informed consent to aid in their defense of a legal claim. In New York, a patient sued after an orthodontist unsuccessfully attempted to bring an impacted canine into occlusion. The patient’s teeth became uneven after the removal of braces, and the patient suffered from root resorption. The orthodontist ultimately won, and the case was dismissed because the orthodontist had a proper informed consent. The orthodontist’s records showed that the patient was orally advised of all the possible side effects of the treatment options, including possible bone loss, unphysiological occlusion, root resorption, ankylosis and the general risks of possible periodontal disease, and that the plaintiff ultimately chose to pursue orthodontic repositioning of the cuspid. The patient records also showed that the patient missed several appointments. Hence, proper records and informed consent aided in the orthodontist’s defense against a claim that included negligence (17).

## Conclusions

• Periodontal issues was the most common reason for a malpractice claim being filed against an orthodontist.

• Doctor-patient rapport was ranked as being the most relevant contributory factor and most relevant mitigating factor in an orthodontic malpractice claim.

• Referring a patient with periodontitis to a periodontist prior to commencing treatment was deemed most relevant in mitigating a malpractice claim.

• It has been ruled in some states that a general dentist can be an expert witness and provide expert testimony against an orthodontist in a lawsuit.
